# Structural analysis of pathogenic missense mutations in *GABRA2* and identification of a novel de novo variant in the desensitization gate

**DOI:** 10.1002/mgg3.1106

**Published:** 2020-04-29

**Authors:** Alba Sanchis‐Juan, Marcia A. Hasenahuer, James A. Baker, Amy McTague, Katy Barwick, Manju A. Kurian, Sofia T. Duarte, NIHR BioResource, Keren J. Carss, Janet Thornton, F. Lucy Raymond

**Affiliations:** ^1^ Department of Haematology University of Cambridge NHS Blood and Transplant Centre Cambridge UK; ^2^ NIHR BioResource Cambridge University Hospitals NHS Foundation Trust Cambridge Biomedical Campus Cambridge UK; ^3^ European Molecular Biology Laboratory European Bioinformatics Institute Wellcome Genome Campus Hinxton, Cambridge UK; ^4^ Department of Medical Genetics Cambridge Institute for Medical Research University of Cambridge Cambridge UK; ^5^ Developmental Neurosciences Great Ormond Street Institute of Child Health University College London London UK; ^6^ Hospital Dona Estefânia Centro Hospitalar de Lisboa Central Lisbon Portugal

**Keywords:** Cys‐loop receptor, epileptic encephalopathy, *GABRA2*, protein structural analysis, whole‐genome sequencing

## Abstract

**Background:**

Cys‐loop receptors control neuronal excitability in the brain and their dysfunction results in numerous neurological disorders. Recently, six missense variants in *GABRA2*, a member of this family, have been associated with early infantile epileptic encephalopathy (EIEE). We identified a novel de novo missense variant in *GABRA2* in a patient with EIEE and performed protein structural analysis of the seven variants.

**Methods:**

The novel variant was identified by trio whole‐genome sequencing*.* We performed protein structural analysis of the seven variants, and compared them to previously reported pathogenic mutations at equivalent positions in other Cys‐loop receptors. Additionally, we studied the distribution of disease‐associated variants in the transmembrane helices of these proteins.

**Results:**

The seven variants are in the transmembrane domain, either close to the desensitization gate, the activation gate, or in inter‐subunit interfaces. Six of them have pathogenic mutations at equivalent positions in other Cys‐loop receptors, emphasizing the importance of these residues. Also, pathogenic mutations are more common in the pore‐lining helix, consistent with this region being highly constrained for variation in control populations.

**Conclusion:**

Our study reports a novel pathogenic variant in *GABRA2*, characterizes the regions where pathogenic mutations are in the transmembrane helices, and underscores the value of considering sequence, evolutionary, and structural information as a strategy for variant interpretation of novel missense mutations.

## Graphical abstract

This study reports a novel pathogenic variant in GABRA2, characterizes the regions where pathogenic mutations are in the transmembrane helices, and underscores the value of considering sequence, evolutionary, and structural information as a strategy for variant interpretation of novel missense mutations.

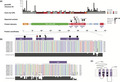

## 1 INTRODUCTION

The dynamic partnership between excitatory principal cells and inhibitory interneurons is essential for proper brain function and needs to be maintained to avoid pathological consequences. Cys‐loop ligand‐gated ion channels are receptors activated by neurotransmitters and play an important role in the development and activity of the central and peripheral nervous systems (Thompson, Lester, & Lummis, 2010). These receptors mediate excitatory and inhibitory synaptic transmissions depending on the distribution of ions at either side of the membrane and the membrane potential of the cell. There are two broad classifications of Cys‐loop receptors, each with different electrophysiological properties: firstly, cation‐selective receptors, corresponding to nicotinic acetylcholine (nACh), serotonin (5‐HT_3_), and zinc‐activated receptors and secondly, anion‐selective members, that include glycine (Gly) receptor and γ‐aminobutyric acid receptors type A (GABA_A_) and C (GABA_C_) (Lester, Dibas, Dahan, Leite, & Dougherty, 2004; Thompson et al., 2010).

Cys‐loop receptors are pentameric channels formed by five chains, each comprising an extracellular domain (where the ligand binds) and a transmembrane (TM) domain consisting of four TM helices (TMH), M1 to M4 (Thompson et al., 2010). The pentamers can be assembled from different gene products that encode different subunits, but all belong to the same type of receptors. For example, for the Cl^−^‐selective GABA_A_ receptors (GABA_A_R), there are 19 genes encoding different subunits (α1‐α6, β1‐β3, γ1‐γ3, ρ1‐ρ3, δ, ε, π, θ) (Gonzalez‐Nunez, 2015; D. D. Wang & Kriegstein, 2009), and the most abundant complex in adult human receptors is a combination of two α, two β, and one γ subunit (Sigel & Steinmann, 2012). Cys‐loop receptors are conserved in humans and across species (Nys, Kesters, & Ulens, 2013; Ortells & Lunt, 1995). Dysfunctions of these receptors demonstrate a critical role in neurological development. Pathogenic variants in genes encoding receptors of nACh (*CHRNA2, CHRNA4*, and *CHRNB2*) and GABA_A_ (*GABRA1, GABRB1, GABRB2, GABRB3, GABRG2,* and *GABBR2*) have previously been associated with human epilepsy (Shinichi Hirose, 2014), and mutations in Gly receptors (*GLRA1* and *GLRB*) cause hyperekplexia (Al‐Owain et al., 2012; Shiang et al., 1993).

For many years, *GABRA2* (MIM: 137,140), that encodes for the GABA_A_R subunit α2 (GABA_A_R α2), remained as a candidate gene for epilepsy. Multiple genome‐wide association studies identified noncoding single‐nucleotide polymorphisms in *GABRA2* associated with increased risk for epilepsy (International League Against Epilepsy Consortium on Complex Epilepsies, 2014, 2018), as well as alcohol dependence and brain oscillations (Edenberg et al., 2004; Strac et al., 2015). Also, decreased expression of *GABRA2* in *Scn1a* ± mice served as a model of Dravet syndrome (Follwaczny et al., 2017; Hawkins, Zachwieja, Miller, Anderson, & Kearney, 2016), and increased expression observed in Pumilio‐2–deficient mice resulted in enhanced seizure susceptibility and the manifestation of epilepsy in the hippocampus (Follwaczny et al., 2017). *GABRA2* is highly expressed in the hippocampus, especially in early development, and is localized to the cell soma to mediate synaptic transmission (Prenosil et al., 2006; Tian, Chen, Cross, & Edenberg, 2005). Recently, six missense variants in *GABRA2* have been reported to cause early infantile epileptic encephalopathy (EIEE) (Butler et al., 2018; Maljevic et al., 2019; Orenstein et al., 2018): five were de novo and one was present in two affected siblings, inherited from a father who also presented the variant at low level in blood, indicating mosaicism.

Here, we describe the seventh variant in *GABRA2* to be reported as de novo in an individual with EIEE identified by whole‐genome sequencing (WGS). We perform protein structural mapping and analysis of all the seven variants in the GABA_A_R α2 and investigate the effect of the novel variant in the protein. We analyze the presence of variants in equivalent positions in other members of the Cys‐loop receptor family and compare their reported effect. Furthermore, because the seven variants cluster in the TM domain, we also analyze the distribution of previously reported pathogenic variants of the Cys‐loop receptors. Our results demonstrate the utility of performing variant interpretation by gathering together sequence, evolutionary, and structural information from homologous Cys‐loop receptors to facilitate the characterization of novel candidate missense variants.

## 2 MATERIALS AND METHODS

### 2.1 Ethical compliance

The proband and both unaffected parents were recruited to the NIHR BioResource research study. The study was approved by the East of England Cambridge South national institutional review board (13/EE/0325). The research conforms with the principles of the Declaration of Helsinki. All participants provided written informed consent to participate in the study.

### 2.2 Genomic analysis

WGS was performed on DNA extracted from whole blood at 30x coverage using Illumina HiSeq X Ten system (Illumina, Inc., San Diego, CA, USA) with 150bp paired‐end reads. Reads were aligned to the human genome of reference GRCh37 using Isaac Aligner, and single‐nucleotide variants and indels were called with both Isaac Variant Caller (Raczy et al., 2013) and Platypus (http://github.com/andyrimmer/Platypus). Variant annotation was performed with Variant Effect Predictor (McLaren et al., 2016), which included allelic population frequencies from gnomAD (release 2.1.1) (Lek et al., 2016) and deleteriousness scores from CADD (Kircher et al., 2014), GERP (Davydov et al., 2010), SIFT (Ng & Henikoff, 2003), and Polyphen‐2 (Adzhubei, Jordan, & Sunyaev, 2013). Structural variants were also called by Manta (Chen et al., 2016) and Canvas (Roller, Ivakhno, Lee, Royce, & Tanner, 2016) algorithms, as described previously (Carss et al., 2017; Sanchis‐Juan et al., 2018). Trio analysis focused on de novo and rare biallelic variant discovery unrestricted by a gene list. Candidate variants were then confirmed by Sanger sequencing.

### 2.3 Protein sequence conservation

Conservation of GABA_A_R α2 was analyzed across the orthologue protein sequences for different model species and across the paralogue Cys‐loop receptors from human. For that, the canonical protein sequences were obtained from UniProtKB (UniProt, 2019), then aligned using MAFFT (Katoh & Standley, 2013) with default parameters. Alignments were visually inspected and manually corrected using JalView (Waterhouse, Procter, Martin, Clamp, & Barton, 2009).

### 2.4 GABA_A_R structural analysis

There is no experimentally determined structure for the human GABA_A_R α2 subunit, either in a homo‐ or hetero‐pentameric complex. For the structural analysis of the previously reported and the novel variants, we obtained from Protein Data Bank (PDB) (Berman et al., 2000) the recently solved cryo‐electron microscopy (cryo‐EM) human hetero‐pentameric GABA_A_R α1β3γ2 complexes in closed (ID 6HUG, with resolution 3.1Å) and desensitized (ID 6HUP and 6I53, with resolution 3.58Å and 3.2Å, respectively) forms (D. Laverty et al., 2019; Masiulis et al., 2019). These structures were used as they represent the most abundant arrangement of adult human GABA_A_R hetero‐pentamers, which is two α subunits, two β subunits, and one γ subunit, with best resolution in PDB. Also, the variants analyzed were in the M1, M2, and M3 segments of the TM domain, and this region was observed, by paired local sequence alignment, to present 100% identical residues between human GABA_A_R α1 and GABA_A_R α2.

The closed‐form structure was solved in complex with the pore blocker picrotoxin and the megabody Mb38 protein. The desensitized structures were captured in complex with diazepam (DZP), GABA, and megabody Mb38 for the ID 6HUP, and only with megabody Mb38 for the ID 6I53.

Structural mapping and visual inspection of the variants in these structures were performed using PyMOL (Delano, 2002). The electrostatic surface visualization was done using its APBS plugin (Baker, Sept, Joseph, Holst, & McCammon, 2001; Dolinsky et al., 2007). Residue interactions were calculated using Residue Interaction Network Generator (RING) (Martin et al., 2011) and visualized also with PyMOL.

Root‐mean‐square deviations (RMSD) were calculated fitting the structures (6HUG, 6HUP, and 6I53) among each other using the McLachlan algorithm (McLachlan, 1982) as implemented in the program ProFit (Martin, A.C.R., http://www.bioinf.org.uk/software/profit/) (Table S4).

### 2.5 Channel pore characterization

To observe the effect of the pore‐lining variants in the pore shape through the ion channel of the GABA_A_R α1β3γ2 structures, four different configurations were considered: (a) where there was no mutated α1 subunit, (b) where the mutated α1 subunit was between β3 and γ2 (α1^β3γ2^), (c) where the mutated α1 subunit was between β3 and β3 (α1^β3β3^), and (d) where both α1 subunits were mutated (α1^β3γ2‐β3β3^). The three mutant structures for each closed and desensitized forms were generated with the *BuildModel* command of FoldX (Schymkowitz et al., 2005), with a previous minimization using *RepairPDB* of the same program. In both steps, the “*membrane”* parameter was turned on, and for *BuildModel*, 20 runs were requested. Then, for each mutant configuration, the structure with the lowest difference in free energy of unfolding (∆∆G=∆G_mutant_‐∆G_WT_) was selected and the radii along the pore axis were calculated in steps of 3Å with PoreWalker (Pellegrini‐Calace, Maiwald, & Thornton, 2009), and compared between them.

### 2.6 Pathogenic variants in TMH of Cys‐loop receptors

We characterized the distribution of pathogenic variants in other Cys‐loop receptor proteins (Table S1). Pathogenic variants were obtained from ClinVar annotated as “Pathogenic,” “Likely pathogenic,” or “Pathogenic/Likely pathogenic” (accessed 31‐Jan‐2019) (Landrum et al., 2018), and Humsavar, annotated as “Disease” (release 13‐Feb‐2019, https://www.uniprot.org/docs/humsavar.txt). Pathogenic variants were mapped to UniProt canonical sequences using VarMap (Stephenson, Laskowski, Nightingale, Hurles, & Thornton, 2019). Only missense variants were considered. Duplicates between Humsavar and ClinVar were only counted once, where a duplicate was considered to be the same amino acid change at the same position in the same protein, and variants with conflicting interpretations between Humsavar and ClinVar were omitted. TRANSMEM annotation by SwissProt was used to determine TMH boundaries.

The Chi‐square test from the SciPy python package (https://docs.scipy.org/doc/scipy/reference/generated/scipy.stats.chisquare.html) was used to compare the observed frequencies of variants that caused disease in M1, M2, M3, and M4 with those expected by chance.

## 3 RESULTS

### 3.1 Clinical evaluation

A female was born at term (41 weeks) by Cesarean section. There was no family history of disease and no consanguinity reported. At delivery, Apgar scores were 9 and 10 and her head circumference was 34 cm (25th‐50th percentile). She presented at 15 months of age with nonspecific EIEE and global hypotonia. Seizures occurred during sleep, with and without fever, and were tonic with upward eye deviation, or eye and head deviation to either side. MRI performed at 19 months was normal. EEG at onset of epilepsy was normal, but later investigations showed slow and irregular background activity, without paroxysmal activity. She presented developmental delay, especially affecting language (comprehension and expression), but also behavior disturbance including hyperactivity, repetitive routines or rituals, and marked disturbance with changes in the environment. She also developed hand stereotypies (waving, finger repetitive movements). She was treated with sodium valproate and clobazam that controlled her seizures. Currently, she is 10 years old and has severe impairment of language, hand stereotypies, disruptive behavior, and repetitive movements. All routine genetic analyses were negative. Additional clinical details for this individual are compared to the previously reported cases (Maljevic et al., 2019) in Table S2.

### 3.2 Identification of a novel de novo mutation in *GABRA2*

**Figure 1:**
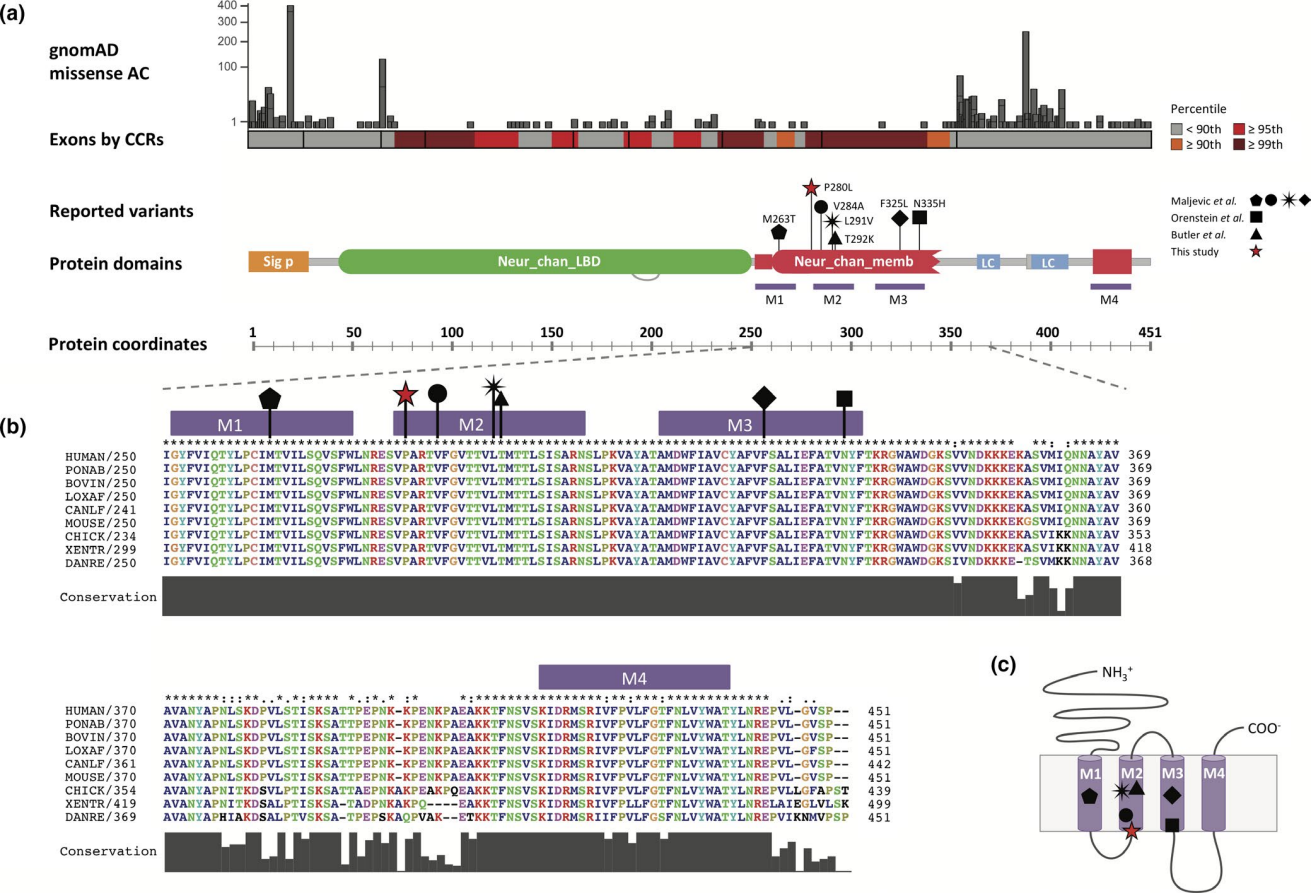
(a) Schematic diagram showing the architecture of domains in GABA_A_ α2. At the top are represented the allele count of missense variants followed by the constrained coding regions’ percentiles (Havrilla et al., 2018), both based on gnomAD release 2.1.1. The seven variants in *GABRA2* are indicated with different symbols (triangle, square, circle, diamond, pentagon, black star, and red star), and cluster in the TM region, which is highly constrained for variation in control population (see also Supp. Figure 2a). Coordinates of the protein domains were obtained from Pfam (https://pfam.xfam.org). Sig_p = signal peptide; Neur_chan_LBD = neurotransmitter‐gated ion‐channel ligand‐binding domain; Neur_chan_memb = neurotransmitter‐gated ion‐channel TM region; LC = low complexity; TM = transmembrane. (b) Alignment of the TM region of GABA_A_ α2 subunit across representative vertebrate species. The regions where the variants fall are evolutionarily conserved through these species. Colors are by amino acid properties. Ponab = orangutan; Bovine = cow; Loxav = elephant; Canlf = dog; Chick = chicken; Xentr = xenopus; Danre = zebrafish. (c) Schematic representation of GABA_A_ α2 TMH. The locations of the seven missense variants are approximate

A novel de novo missense mutation was identified in *GABRA2* at genomic position Chr4(GRCh37):g.46305494 G > A, NM_000807.2:c.839C > T, NP_000798.2:p.Pro280Leu by WGS trio analysis of DNA from both parents and the child. The variant was confirmed by Sanger sequencing (Figure S1). No other candidate variant was identified in this individual from trio analysis. The gene was observed to be constrained for loss of function (LOF) variation (with pLI = 1 and observed/expected score = 0.05) and missense variation (Z score = 3.13) in gnomAD (Lek et al., 2016), especially in the TM region and ligand‐binding domain of the neurotransmitter‐gated ion channel (Figure 1a and Figure S2) (Havrilla, Pedersen, Layer, & Quinlan, 2018).

Pro280 is located in M2, the second helix in the TM domain (Figure 1b,c). It is highly conserved across species (Figure 1b) and across different subunits from the GABA_A_R family in human (Figure S3a). This variant is absent in gnomAD database (Figure 1a) and was predicted to be damaged by CADD, GERP, SIFT, and Polyphen‐2 (with scores of 29.4, 5.17, 0.02 and 1, respectively).

### 3.3 Structural characterization of the novel and previously reported variants in GABA_A_R α2

Protein structural mapping of the seven variants revealed their proximity to the desensitization gate, the activation gate, or the M1‐M3 inter‐subunit interfaces of the receptor.

**Figure 2:**
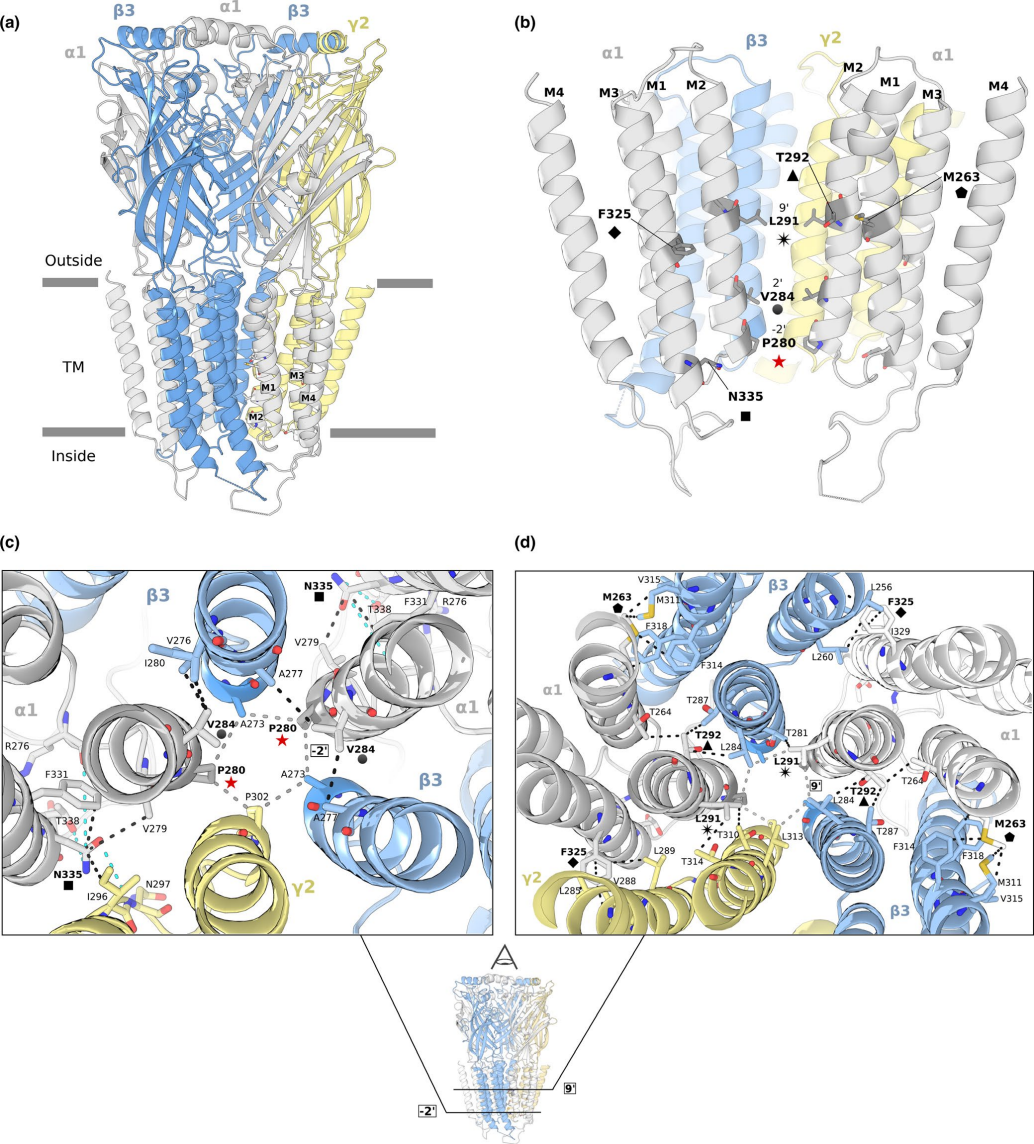
(a) The GABA_A_ α1β3γ2 receptor in closed state. M1 to M4 helices are depicted over the homologous human GABA_A_R α1 chain (PDB ID 6HUG). The two α1 chains are in grey, the two human β3 chains in blue, and the human γ2 in yellow, with the approximate boundaries of the TM in grey bars. (b) Side view of the transmembrane helices of the GABA_A_ α1β3γ2 receptor in closed state. The structural location of the variants is depicted consistently using the same symbols as in Figure 1a. For a clearer view, the chain between two α1 subunits is hidden. (c) Down the pore axis view of the variants nearby the −2’ ring (Pro280Leu, Val284Ala, Asn335His), and (d) the 9’ ring (Met263Thr, Leu291Val, Thr291Lys, Phe325Leu). VDW interactions for the residues where the variants fall are in black dashed lines (except for the rings that are in grey) and hydrogen bonds are in cyan

**Figure 3:**
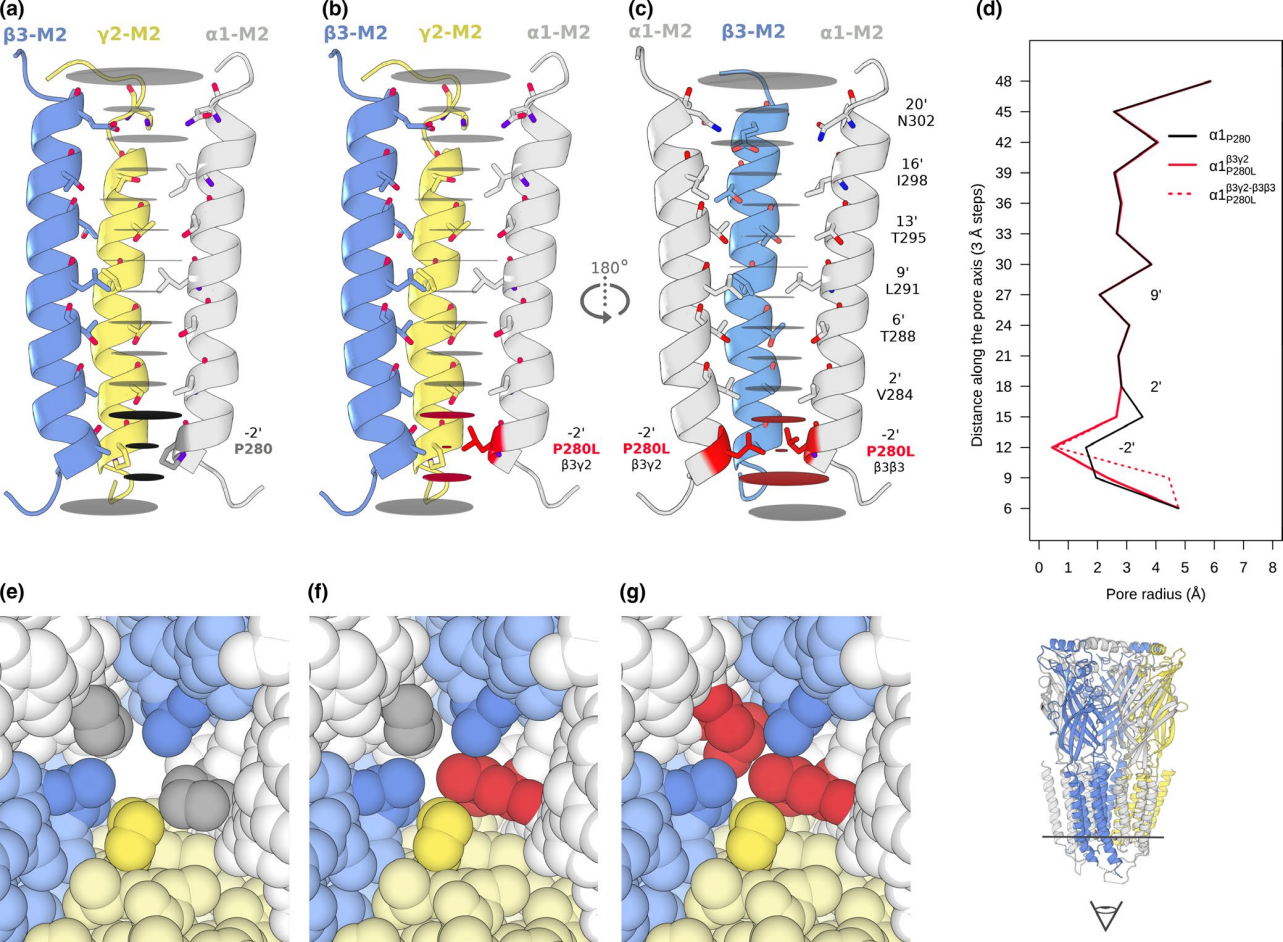
Effect of Pro280Leu in the pore radii of the GABA_A_ α1β3γ2 receptor in its closed form. Radii profile through the TM pore axis by 3 Å steps for: (a) Nonmutant GABA_A_ α1β3γ2 (α1_P280_), (b) mutant α1 subunit is between β3γ2 (α1^β3γ2^_P280L_), and (c) both α1 subunits are mutated (α1^β3γ2‐β3β3^_P280L_). M2 pore‐lining residues are shown in sticks. The pore radii at each step along the pore vertical axis are represented with horizontal grey discs. The pore is narrowest in α1_P280_ at the −2’ position (1.60 Å), which is smaller than the Pauling ionic radius of Cl^−^ (1.81 Å) and the radius of hydrated Cl^−^ (3.2 Å). For clarity, only three M2 helices are represented in each situation. The steps with affected radii upon Pro280Leu are colored in red in (b) and (c). (d) Pore radius plotted as a function of longitudinal distance along the pore axis, vertically aligned to match the steps in (a), (b), and (c) panels. The biggest radii reduction can be observed at 12 Å (−2’ position) from 1.60 Å in α1_P280_ to: 0.58 Å (∆=1.02 Å) in α1^β3γ2‐β3β3^_P280L_ and 0.45 Å (∆=1.15 Å) in α1^β3γ2^_P280L_. Also, reductions can be observed at 15 Å (region between −2’ and 2’ positions) from 3.55 Å in α1_P280_ to: 2.64 Å (∆= 0.91 Å) in α1^β3γ2‐β3β3^_P280L_ and 2.64 Å (∆= 0.91 Å) in α1^β3γ2^_P280L_. Increments in the radii are observed at 9 Å from 1.95 Å in α1_P280_ to: 4.44 Å (∆=2.49 Å) in α1^β3γ2‐β3β3^_P280L_ and 2.33 Å (∆=0.38 Å) in in α1^β3γ2^_P280L_. This step at 9 Å corresponds to the vestibule region adjacent to the −2' ring in the inner cytoplasmic side. Zoom over the inner cytoplasmic vestibule of the pore defined by the amino acids of the −2’ ring for (e) nonmutant GABA_A_ α1β3γ2, (f) mutant α1 subunit is between β3γ2 (α1^β3γ2^_P280L_), and (g) both α1 subunits are mutated (α1^β3γ2‐β3β3^_P280L_). The color for each subunit chain matches the ones used in Figure 2

The variants at the desensitization gate are Pro280Leu and Val284Ala. This gate is defined by the region between −2’ and 2’ positions of the receptor and modulates the conductance and ion‐selectivity, as observed in the structures of GABA_A_ and Gly receptors (Gielen, Thomas, & Smart, 2015; Hibbs & Gouaux, 2011; Sauguet et al., 2013). Pro280 is located at the TM region of the receptor, in the N‐terminal end of the M2 helix in the cytoplasmic facing leaflet (Figure 2), and corresponds to the exact −2’ position. The side chains of the amino acids in this position (Pro in the case of α1‐α6 and γ1‐γ3 subunits, Ala in β1‐β3 subunits), coming from each chain of the pentamer, altogether define the inner narrowest constriction of the pore, known as the −2’ ring (Hibbs & Gouaux, 2011), as shown in Figure 2b and c. A reduction in the diameter of the pore is observed upon the introduction of this variant (Figure S4a,b). Small nonpolar amino acids (proline and alanine) are conserved at the −2’ position in human GABA_A_ and Gly anion‐selective receptors (Figure 3a), suggesting the necessity of maintaining the size, shape, and absence of charge in the inner constriction of this pore for the adequate transit of anions. In contrast, in cation‐selective receptors, a negatively charged amino acid (glutamate) with a longer and more flexible side chain is usually observed in position −1’ (Figure S3a).

Val284, corresponds to the precise 2’ position. In this case, the side chains of the amino acids in this position do not define a ring, but establish inter‐subunit Van der Waals (VDW) interactions with M2 helices from the β3 subunits (Figure 2c) (Hibbs & Gouaux, 2011). Having an alanine instead of a valine at this position affects the radius of the pore differently depending on the arrangement of the different subunits and the state of the receptor (Figure S4a,c).

The variant Asn335His is near the desensitization gate, at 9.5 Å (in PDB ID 6HUP) from −2’ position in α1‐M2 (Pro280). Asn335 is located at the C‐terminus of the M3 helix, on the inner side of the TM region, at the base of the helical bundle, and participates in inter‐ and intra‐subunit interactions with residues in the M2‐M3 loops of β3 and γ2 chains, including Val279 (Figure 2c). This network of contacts has been proposed to be important to stabilize the desensitized state of a GABA_A_R (the homo‐pentameric GLIC‐GABA_A_R α1 chimeric receptor) (Gielen et al., 2015; Duncan Laverty et al., 2017). This stabilization could be affected by the substitution to a positively charged histidine.

The variant Leu291Val is at the activation gate, which has been defined as the ring formed by the leucines at the 9’ position, and is important for the transition between opened and closed states of the ion channel (Duncan Laverty et al., 2017). The precise 9’ position corresponds with Leu291 and is located in the pore‐lining M2 helix of the TM domain (Figure 2b,d). Having a valine instead of a leucine at this residue increases the diameter of the pore (Figure S4a,d) and alters the VDW interactions that define the 9’ ring as well as the inter‐subunit interactions with the threonines from the neighboring β3 and γ2 chains.

Thr292Lys is at the 10’ position, which is adjacent to the activation gate. Thr292 is at the turn of the alpha helix that faces the inter‐subunit interface, and is involved in inter‐ and intra‐subunit VDW interactions with Thr264 in M1 of its same α1 subunit, and with the β3 subunit residues Leu287 and Leu284, being the last one part of the 9’ ring of this subunit (Figure 2b,d).

The variants in M1‐M3 inter‐subunit interfaces are Met263Thr and Phe325Leu. Met263 is located at the middle of the M1 helix, at 13.5Å (in PDB ID 6HUP) from the 9’ activation gate of β3‐M2. It is involved in a network of VDW interactions with residues from the M3 helix of the neighboring β3 subunit, and it is located near the Thr264 residue, which interacts with the Thr292 (Figure 2b,d). Met263 is also part of the recently reported low‐affinity binding site for benzodiazepines, a group of sedative anticonvulsant drugs (Masiulis et al., 2019; Olsen, 2018; Walters, Hadley, Morris, & Amin, 2000). In the desensitized GABAA α1β3γ2 receptor bound to diazepam (DZP), Met263 is part of the opening of the pocket to the external molecular surface and its lateral chain sulfur establishes direct interaction with the diazepine ring of DZP (Figure S5). Having a smaller threonine with a polar uncharged lateral chain instead of methionine would affect the size and shape of the entrance to this pocket and the interactions established with the benzodiazepines. It can also be observed, deeper in the pocket, that the benzene ring of DZP also interacts with Thr292.

Lastly, Phe325 is at the middle of M3 helix, at 3.7 Å (in PDB ID 6HUP) from the 9’ activation gate of α1‐M2. It is also involved in a network of VDW interactions with residues of M1 from either β3 or γ2 (Figure 2b,d). Some evidence points to the α1 residues Ser297 and Ala318 in the α1‐M3 β3‐M2 TM interface as critical for the receptor modulation by diverse anesthetic molecules (Mihic et al., 1997).

### 3.4 The novel variant Pro280Leu affects the diameter of the channel pore in the desensitization gate

We introduced in silico the mutation Pro280Leu into the cryo‐EM structure of GABA_A_R α1β3γ2 closed and desensitized forms (Figure 3 and Figure S4), in the four situations: α1_P280_, α1^β3γ2^_P280L_, α1^β3γ2‐β3β3^_P280L_, and α1^β3β3^_P280L_. In Figure 3, only the first three are shown for simplicity.

The larger nonpolar amino acid chain of the leucine narrowed the constriction region compared to the nonmutated receptors (Figure 3d and Figure S4a,b) measuring the radius at 3 Å steps along the pore axis. The largest changes in all the situations occurred at 12 Å (−2’) and 15 Å. For the desensitized structure of the receptor that has GABA and DZP, this reduction can also be observed at 18 Å (2’).

FoldX stability calculations showed higher ∆∆G values, which correspond with higher destabilizing effects in the desensitized compared to the closed forms (Table S3). Also, these showed higher ∆∆G values when both α1 chains were mutated.

### 3.5 Location of GABA_A_α2 mutations is equivalent to pathogenic variants in other Cys‐loop receptors

We investigated if the location of the seven variants in GABA_A_α2 had equivalent positions also mutated and associated with disease in other Cys‐loop receptors. Six of the seven variants had at least one pathogenic mutation at the equivalent or flanking position in other Cys‐loop receptor genes. These variants are highlighted in the alignment of the Cys‐loop receptor sequences in Figure S3b. Additional information for the variants is shown in Table 1.

**Table 1:** Details of variants in equivalent positions to the seven mutations in *GABRA2* (NM_000807.2) identified in other Cys‐loop receptor genes

**Mutation in *GABRA2***	**Gene**	**Family**	**HGVSc**	**HGVSp**	**Equivalent position in *GABRA2***	**Reference SNP**	**References**	**Source**
**Met263Thr**	*CHRNA2*	nACh	NM_000742.3(CHRNA2):c.836T > A	Ile279Asn	262	rs104894063	(Aridon et al., 2006)	ClinVar; Humsavar
	*GABRA1*	GABA‐A	NM_000806.5(GABRA1):c.788T > A	Met263Lys	263	rs796052491	–	ClinVar
	*GABRA1*	GABA‐A	NM_000806.5(GABRA1):c.789G > A	Met263Ile	263	rs1060499553	(Farnaes et al., 2017)	ClinVar
	*GABRA1*	GABA‐A	NM_000806.5(GABRA1):c.788T > C	Met263Thr	263	–	(Kodera et al., 2016)	Manual
	*GABRB3*	GABA‐A	NM_000814.5(GABRB3):c.766C > G	Leu256Val	263	rs1555401942	–	ClinVar
	*GABRB3*	GABA‐A	NM_000814.5(GABRB3):c.767T > A	Leu256Gln	263	–	(Epi, 2016)	Humsavar
**Pro280Leu (−2’)**	*GABRG2*	GABA‐A	NM_198903.2(GABRG2):c.905C > T	Pro302Leu	280	–	(Hernandez et al., 2017)	Manual
	*GLRA1*	Gly	NM_001146040.1(GLRA1):c.832C > A	Pro278Thr	280	rs121918413	(Saul et al., 1999)	ClinVar; Humsavar
**Val284Ala (2’)**	*CHRNA2*	nACh	NM_000742.3(CHRNA2):c.889A > T	Ile297Phe	283	rs1554514507	(Conti et al., 2015)	ClinVar
	*GABRB2*	GABA‐A	NM_021911.2(GABRB2):c.830T > C	Leu277Ser	285	rs1554094145	(Hamdan et al., 2017)	ClinVar
	*GABRG2*	GABA‐A	NM_000816.3(GABRG2):c.919T > G	Leu307Val	285	rs796052509	–	ClinVar
**Leu291Val (9’)**	*CHRNB1*	nACh	NM_000747.2(CHRNB1):c.853C > A	Leu285Met	291	rs137852811	(Gomez et al., 1996)	ClinVar; Humsavar
	*GLRB*	Gly	NM_000824.4:c.920_921ΔInsGA	Leu307Arg	291	–	(James et al., 2013)	Humsavar
	*GABRA1*	GABA‐A	NM_000806.5(GABRA1):c.868G > A	Val290Met	290	rs796052494	–	ClinVar
	*GABRB2*	GABA‐A	NM_021911.2(GABRB2):c.845T > C	Val282Ala	290	rs886039374	–	ClinVar
	*GLRA1*	Gly	NM_001146040.1(GLRA1):c.862G > A	Val288Met	290	rs121918416	(del Giudice et al., 2001)	ClinVar
**Thr292Lys**	*GLRA1*	Gly	NM_000171.3(GLRA1):c.869C > T	Thr290Ile	292	rs1064795411	–	ClinVar
	*CHRNA4*	nACh	NM_000744.6(CHRNA4):c.851C > T	Ser284Leu	292	rs28931591	(Cho et al., 2003; S. Hirose et al., 1999; Hwang et al., 2011; Kurahashi & Hirose, 2002)	ClinVar
**Phe325Leu**	*CHRNB2*	nACh	NM_000748.2(CHRNB2):c.923T > C	Val308Ala	324	rs281865070	–	ClinVar
	*GABRA1*	GABA‐A	NM_000806.5(GABRA1):c.973T > C	Phe325Leu	325	rs1064794681	–	ClinVar
	*GABRB2*	GABA‐A	NM_021911.2(GABRB2):c.946G > A	Val316Ile	324	rs1554093884	–	ClinVar
	*GABRB2*	GABA‐A	NM_021911.2(GABRB2):c.950_951delTCinsCA	Phe317Ser	325	rs1554093882	–	ClinVar
	*CHRNA1*	nACh	NM_001039523.2(CHRNA1):c.988G > A	Val330Ile	324	rs137852804	(H. L. Wang et al., 1999)	ClinVar

The same amino acid change that we observed in *GABRA2* (Pro280Leu) was reported at the equivalent location in *GABRG2* in an individual with Dravet syndrome (NM_198903.2(*GABRG2*):p.Pro302Leu) (Hernandez et al., 2017), and a different amino acid change was observed in an individual with autosomal dominant hyperekplexia (NM_001146040.1(*GLRA1*):p.Pro278Thr) (Saul et al., 1999). Both mutations, like the Pro280Leu, were in the −2’ ring of the receptors. The authors demonstrated that these mutations enhanced desensitization and reduced both the “channel open” probability and the frequency of receptor single‐channel openings.

For the Met263, five pathogenic mutations in other Cys‐loop receptor genes were observed to be equivalent to this exact position, and one was present in the adjacent amino acid (Aridon et al., 2006; Farnaes et al., 2017; Kodera et al., 2016; Epi, 2016). This variant is in the M1 helix of the TM domain, in the inter‐subunit interface with the M3, and this region is important for the actions of potentiating neuroactive steroids (Akk et al., 2008). Functional studies of the variant equivalent to the previous amino acid in *CHRNA2* (NM_000742.3:p.Ile279Asn) showed that it increases the receptor sensitivity to the neurotransmitter, without affecting desensitization properties and channel permeability.

Three variants reported in the literature were observed in the adjacent amino acids to the Val284Ala (2’) of *GABRA2* (Table 1) (Conti et al., 2015; Hamdan et al., 2017). Functional studies on the NM_000742.3(*CHRNA2*):p.Ile297Phe were consistent with the loss of function of the receptor, either by impaired channel expression onto the cell surface or by a drastic decrease in the channel open probability (Conti et al., 2015).

Variants in the leucines that form the 9’ ring have been previously reported in other members of the Cys‐loop receptors as associated with congenital myasthenic syndrome and hyperekplexia (Table 1) (Gomez et al., 1996; James et al., 2013). Mutations at this site are known to destabilize the closed state of the receptor and produce spontaneously active channels. Functional works on the NM_000747.2(*CHRNB1*):p.Leu285Met (9’) showed reduced stability of the closed gate and abnormal channel openings even in the absence of the neurotransmitter, consistent with a receptor that is caught in the open state (Gomez et al., 1996).

The equivalent position to Thr292 in GABA_A_ α2 has been reported as pathogenic in *GLRA1* (NM_000171.3:p.Thr290Ile) and *CHRNA4* (NM_000744.6:p.Ser284Leu) (Cho et al., 2003; S. Hirose et al., 1999; Hwang, Makita, Kurahashi, Cho, & Hirose, 2011; Kurahashi & Hirose, 2002). Expression studies of the *CHRNA4*‐Ser284Leu demonstrated higher affinity to acetylcholine and faster desensitization of the receptors and is reported in multiple affected individuals (Steinlein, Hoda, Bertrand, & Bertrand, 2012).

Lastly, two variants were reported at the equivalent position to Phe325 in *GABRA2* and three were in the adjacent amino acid (Table 1). Functional studies on the variant in *CHRNA1* (NM_001039523.2:p.Val330Ile, equivalent to the adjacent Val324 in *GABRA2*) showed it affects the speed and efficiency of gating of its channel, slowing opening and increasing closing rates (H. L. Wang et al., 1999).

### 3.6 Disease variants in TMH of Cys‐loop receptors are most commonly found in M2

The previously reported and novel variants in *GABRA2* fall in the TMH of the ion channel. TMHs are enriched for germline disease variants compared to other regions (Dobson, Meszaros, & Tusnady, 2018). Therefore, we investigated the location of all pathogenic ClinVar and Humsavar variants reported in proteins from the Cys‐loop receptors (Table S2) to contain pathogenic variants present in ClinVar and Humsavar.

**Figure 4:**
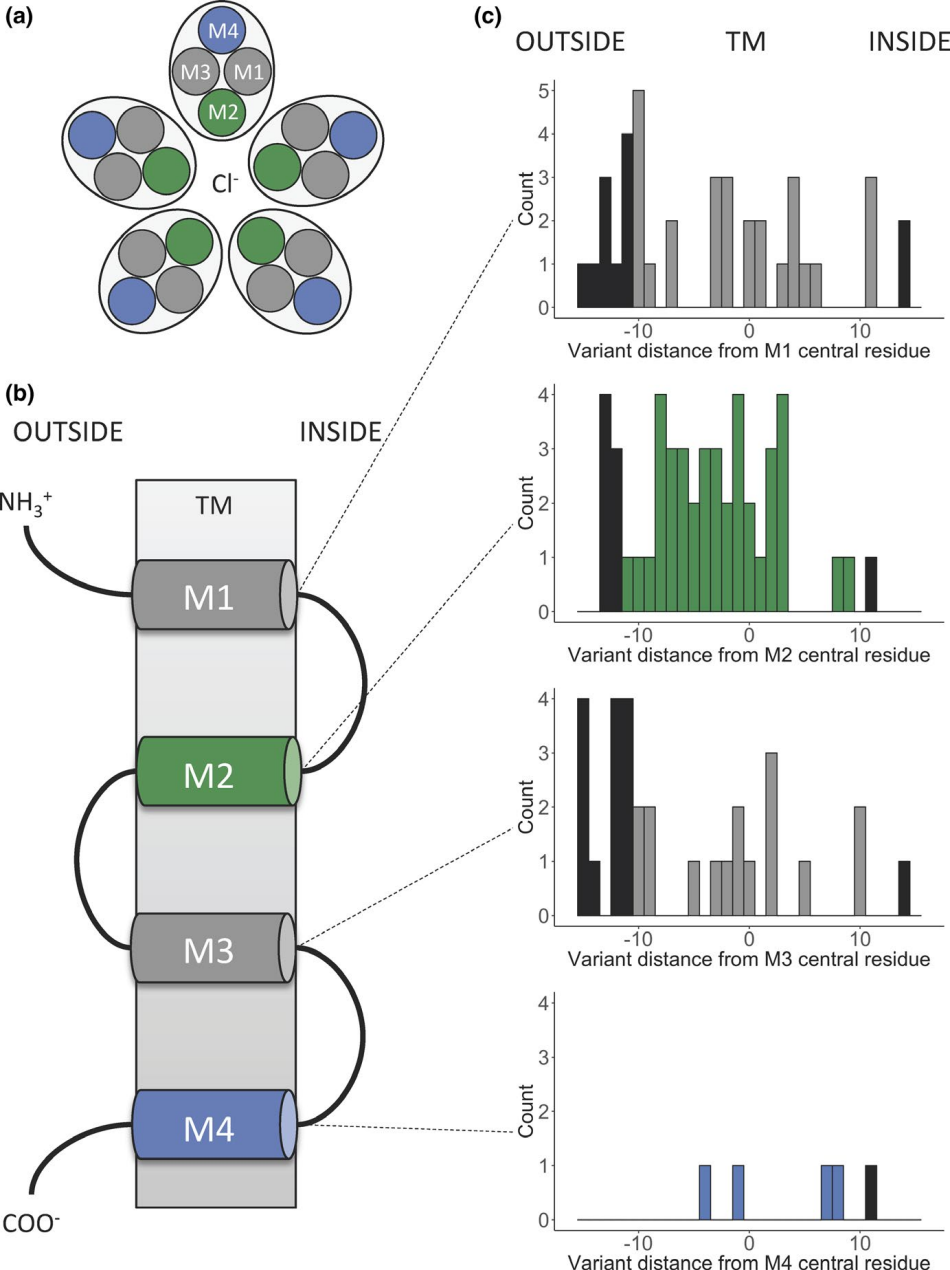
Distribution of disease variants along the TMH of Cys‐loop receptors. (a) Top view schema of the TMH arrangement, considering the five subunits. (b) Simplified linear schema of the four helices of one subunit in the TM. (c) Positions of disease variants plotted as a distance in residues from the central TM residue in sequence. Negative positions are toward the “outside” (extracellular space), whereas positive positions are toward the “inside” (cytosol). The number of variants observed in the TMH was 86: M1 = 27, M2 = 39, M3 = 16, M4 = 4, and 122 including ± 5 flanking residues: M1 = 39, M2 = 47, M3 = 31, M4 = 5. In the histograms, disease variants in TMH are colored according to their position in the schema from Figure 4b, and disease variants in flanking regions are colored black. Duplicates in ClinVar and Humsavar are counted as one variant

About 265 variants were within the TMH, of which 86 were annotated as disease‐causing. Three hundred and ninety‐eight variants were found in the TMH including ± 5 flanking residues, of which 122 were annotated as disease‐causing, being 82 from ClinVar, 11 from Humsavar, and 29 from both. Within the TMH region, disease variants were more commonly found in, or in close proximity to, M2 than other TMH. Thirty‐nine disease variants were observed in the M2 helix itself, and 47 when considering the ± 5 flanking amino acids. M2 was the most populated helix for disease variants compared to M1, M3, and M4 (χ^2^ test p‐value = 3.8e‐7), and this effect was similar considering the TMH including the ± 5 flanking residues (χ^2^ test p‐value = 7.3e‐7) (Figure 4).

Notably, as a trend, the variants in M2 were distributed throughout the helix, whereas in M1, which is the second most populated helix for disease variants, these were most common at the lipid‐water interface and flanking regions particularly on the extracellular side (a peak of five disease variants at position −10. Also, the M1 has nine positions with no observed disease variants compared to four positions with no disease variants in the M2 (Figure 4c). M4 sustained fewer pathogenic variants, consistent with the lower conservation and higher tolerance for variation in gnomAD (Figure 1a, Figure S2).

## 4 DISCUSSION

Here we report a novel de novo missense mutation in *GABRA2*. The phenotype of this case was EIEE, developmental delay, significant hypotonia, and congenital nystagmus, similar to the previously reported individuals. The variant (Pro280Leu) was present in the −2’ position of the channel pore (Bali & Akabas, 2007). This position is important for the ion‐selectivity and desensitization of the ion channel, which are fundamental properties of most ligand‐gated ion channels to change the conformation and limit the ion current flow, despite the neurotransmitter still being bound to the receptor (Gielen et al., 2015). We observed that the variant was in a highly conserved residue and the radius of the pore was narrowed at the gate in both closed and desensitized structures of the receptor. Having a residue like proline with a rigid side chain (or an alanine, observed in the β subunits, with a nonrigid but very small side chain) at the intracellular −2’ position allows tight control of the gating upon neurotransmitter extracellular binding, exerting rigid body motion of the TMH, as can be observed in recent GABA_A_ and Gly receptor structures (Hassaine et al., 2014; Hibbs & Gouaux, 2011; Duncan Laverty et al., 2017; Masiulis et al., 2019). Replacing the constraining proline with a leucine will permit changes in the backbone conformation (von Heijne, 1991). Moreover, ion‐selectivity is determined by the −2’ Pro in anion‐selective channels, along with the −1’ Ala (Keramidas, Moorhouse, Schofield, & Barry, 2004; Thompson et al., 2010), with no requirement for a charged side chain in the narrowest constriction.

Pathogenic missense mutations in *GABRA2* have been suggested to be compatible with both loss and gain‐of‐function (GOF) of the ion channel. Functional experiments on previously reported variants suggested to be compatible with a LOF (Maljevic et al., 2019), while a mutation in the activation gate (9’) was associated with predominantly opened channels (Butler et al., 2018), therefore suggesting to result in the GOF of the receptor. The fact that opposing molecular effects result in the same disease could be explained by the dual role that *GABRA2* presents during development (Jenkins & Escayg, 2019), since early in life, GABA_A_ receptors mediate excitation by depolarizing immature neurons in response to activation by GABA. The function of these receptors changes as the mature Cl^−^ gradient is established later in life, mediating inhibition by exerting hyperpolarization.

Mapping of the seven variants in *GABRA2* on the protein structure showed that they are located at key regions of the receptor. Six of the seven variants identified in *GABRA2* had equivalent pathogenic mutations in other Cys‐loop receptors reported in the literature (Table 1), highlighting the importance of these regions. Although distinct phenotypes associated with the variants in Cys‐loop receptors reflect their unique biological role, this family share a common structural topology conserved throughout evolution. Here, we show how variant interpretation by gathering together sequence, evolutionary, and structural information from homologous Cys‐loop receptors can facilitate the characterization of novel candidate missense variants, especially in those cases where pathogenic mutations have already been characterized.

Furthermore, analysis of the distribution of previously reported pathogenic variants in the Cys‐loop receptors showed that mutations are more common in the M2 segment than any other TMH, consistent with the importance of this helix in shaping the pore along the TM region. Also, the distribution of variants in M2 occurs throughout the helix, while disease variants in other helices that are more distant from the axis of the pore favor the water‐lipid interface regions rather than the center of the TMH. Other clusters of pathogenic variants were observed in the M1 and M3 helices. M1 and M3 are more distant from the axis of the pore but play an important role in defining the inter‐subunit interaction interfaces (Ducan Laverty et al., 2017). In contrast, M4 has fewer pathogenic variants, consistent with this region being less conserved and more tolerant to variation in gnomAD (Figure 1 and Figure S2). Although mutations in these helices have been studied and demonstrated to influence channel gating kinetics, agonist sensitivity, and create spontaneous opening channels in different members of the Cys‐loop receptors (Chang & Weiss, 1999; Chang, Wu, Zhang, & Huang, 2009), their distribution in the protein structure across the different members of the family has never been assessed. Our results provide a better understanding of the structural location of pathogenic variants in the TMH of Cys‐loop receptors, facilitating further interpretation of novel candidate disease‐causing mutations.

Herein, we present a novel de novo missense variant in *GABRA2* in a patient with EIEE and perform protein structural analysis of the previously reported and the novel variants. Our results highlight the importance of performing structural analysis of missense mutations in *GABRA2,* in order to provide a more accurate insight into the etiology of the disease that might also lead to opportunities for personalized treatments.

## CONFLICT OF INTEREST STATEMENT

The authors declare that they have no competing interests.

## AUTHOR CONTRIBUTIONS

ASJ, KJC, and FLR designed the study. ASJ and KJC performed sequencing data processing. ASJ and MAH performed the former analysis and investigation, under the supervision of FLR and JT. JAB performed the statistical analysis. AMT, KS, MK, and STD recruited the family and collected the clinical data and sample. KB performed the Sanger sequencing confirmation of the novel variant. ASJ, MAH, FLR, and JAB wrote the manuscript. All authors read and approved the final manuscript.

## Data Availability

Sequence data for the trio have been deposited at the European Genome‐Phenome Archive (EGAD00001004522).
